# Longevity-Associated Variant of BPIFB4 Confers Neuroprotection in the STHdh Cell Model of Huntington Disease

**DOI:** 10.3390/ijms232315313

**Published:** 2022-12-05

**Authors:** Monica Cattaneo, Anna Maciag, Maria Serena Milella, Elena Ciaglia, Antonino Bruno, Annibale Alessandro Puca

**Affiliations:** 1 Cardiovascular Department, IRCCS MultiMedica, 20138 Milan, Italy; 2 Department of Medicine, Surgery and Dentistry “Scuola Medica Salernitana”, University of Salerno, 84081 Salerno, Italy; 3 Laboratory of Innate Immunity, Unit of Molecular Pathology, Biochemistry and Immunology, IRCCS MultiMedica, 20138 Milan, Italy; 4 Laboratory of Immunology and General Pathology, Department of Biotechnologies and Life Sciences (DBSV), University of Insubria, 20138 Varese, Italy

**Keywords:** mutant Huntingtin, Huntington disease, nucleolar stress, DNA damage, apoptosis and heterochromatin

## Abstract

Huntington’s disease (HD) is caused by the production of mutant Huntingtin (mHTT), characterized by long polyglutamine repeats with toxic effects. There are currently no clinically validated therapeutic agents that slow or halt HD progression, resulting in a significant clinical unmet need. The striatum-derived STHdh cell line, generated from mHTT knock-in mouse embryos (STHdh^Q111/Q111^), represents a useful model to study mechanisms behind pathogenesis of HD and to investigate potential new therapeutic targets. Indeed, these cells show susceptibility to nucleolar stress, activated DNA damage response and apoptotic signals, and elevated levels of H3K9me3 that all together concur in the progressive HD pathogenesis. We have previously shown that the adeno-associated viral vector-mediated delivery of the *longevity-associated variant (LAV) of BPIFB4* prevents HD progression in a mouse model of HD. Here, we show that LAV-BPIFB4 stably infected in STHdh^Q111/Q111^ cells reduces (i) nucleolar stress and DNA damage through the improvement of DNA repair machinery, (ii) apoptosis, through the inhibition of the caspase 3 death signaling, and (iii) the levels of H3K9me3, by accelerating the histone clearance, via the ubiquitin–proteasome pathway. These findings pave the way to propose LAV-BPIFB4 as a promising target for innovative therapeutic strategies in HD.

## 1. Introduction

Huntington’s disease (HD) is an autosomal-dominant neurodegenerative disorder characterized by progressive motor, cognitive, and psychiatric symptoms that typically emerge in midlife. The mutant gene product contains an elongated stretch of CAG repeats in the first exon of the Huntingtin (HTT) gene that translates into an extended sequence of polyglutamines (polyQ) near the N terminus of the HTT protein [1].

The most striking neuropathology is a massive loss of medium-sized spiny neurons (MSNs), which account for more than 90% of the striatal neuronal population [2]. Despite the known genetic cause, the mechanisms of MSN degeneration in HD remain unclear.

The current hypothesis in HD is that neuronal degeneration results from the combined effects of a gain of function in the mutated form of HTT (mHTT) along with a loss of function in the wild-type protein. Indeed, CAG repeat/polyQ expansion confers novel deleterious characteristics on mHTT that include the production of polyQ-bearing N-terminal fragments able to misfold and form detectable aggregates responsible for several aspects of HD [3]. In addition, the gain of the beneficial and neuroprotective effects of wild-type HTT can shield neurons against mHTT toxicity [4]. Consistently, wild-type HTT is important for cell health and viability and its depletion in the mouse central nervous system leads to aberrant synaptic connectivity, cellular stress, neuroinflammation, and neuronal death [5,6,7].

Beyond the reportedly canonical pathological pathways, several studies emphasized the contribution of expanded CAG mRNA itself in HD neurotoxicity. This mechanism accounts for the sequestration, via expanded *CAG* RNA foci, of the RNA binding protein nucleolin (NCL), a nucleolar regulator of ribosomal (r) RNA synthesis [8], resulting in the loss of NCL function. As a consequence, ribosome biogenesis is severely altered, leading to the activation of nucleolar stress response and, ultimately, to cell death through apoptosis [9].

Moreover, transcriptional dysregulation, due to aberrant chromatin remodeling, is emerging as an important driver for the pathogenesis of HD. In HD patients and in vitro and in vivo models, mHTT reportedly impacts transcription and nucleosome dynamics [10] through the heterochromatin condensation via hypermethylation on lysine 9 of H3 (H3K9) [10,11]. Importantly, epigenetic modification by small molecules resolved heterochromatin condensation and the neuropathological phenotype supporting the application of these epigenetic therapeutics in future human clinical trials [10,11,12].

In addition, DNA strand break (DSB) accumulation, atypical ataxia telangiectasia-mutated (ATM) pathway activation and DNA repair dysfunction are reported to be critical contributors in HD-related neuronal dysfunction [13,14,15], indicating novel routes for a therapeutic development in HD.

Bactericidal/permeability-increasing fold-containing family B member 4 (BPIFB4) is a member of the BPI family whose role has been deeply investigated. Its locus, characterized by several single nucleotide polymorphisms (SNPs), is under positive and balancing selection [16]. Among them, four SNPs (rs2070325, rs2889732, rs11699009, and rs11696307) are missense changes in linkage disequilibrium that confer new functions to the protein [17]. The resultant 4-SNPs haplotype distinguishes the wild-type (WT) Ile229/Asn281/Leu488/Ile494-BPIFB4 isoform (allele frequency, 66%) from the longevity-associated variant (LAV) Val229/Thr281/Phe488/Thr494-BPIFB4 (allele frequency, 29.5%) and the rare variant (RV) Ile229/Asn281/Phe488/Thr494-BPIFB4 (allele frequency, 4%). The homozygosity for the minor allele of these four SNP haplotypes is enriched in exceptionally long-living individuals, in agreement with a protective role of this variant [17,18]. Carriers of the LAV allele have higher BPIFB4 circulating protein levels and homozygous for the minor allele display increased levels of phosphorylated endothelial nitric oxide synthase (eNOS) in mononuclear cells [19]. LAV-BPIFB4 gene therapy transduction promotes reparative vascularization and reperfusion in a murine model of peripheral ischemia [17], halts the progression of atherosclerosis and inflammation in ApoE knockout mice [19], rescues diabetic cardiomyopathy in obese mice with type 2 diabetes [20], improved frailty indices in aging mice [21], prevents neuropathological phenotypes in HD models [22] and rejuvenates the immune system and vasculature in aged mice [23]. The mechanisms behind the LAV-BPIFB4 capacity to improve disease resistance are in part explained by its ability to potentiate nitric oxide production by eNOS via calcium mobilization [24] and to induce M2 macrophage polarization, downstream of stromal cell-derived factor 1 (SDF-1/CXCl12)-CXCR4 signaling [19,20,22].

Here, we show that ectopic LAV-BPIFB4 antagonizes the neurotoxicity in the striatal precursor cell line expressing expanded HTT and, to a greater extent, in the neuron-like counterpart. Furthermore, we provide new promising molecular mechanisms behind the neuroprotective effect of the LAV-BPIFB4.

## 2. Results

### 2.1. BPIFB4 Isoforms Confer Resistance to Nucleolar Stress

**Figure 1:**
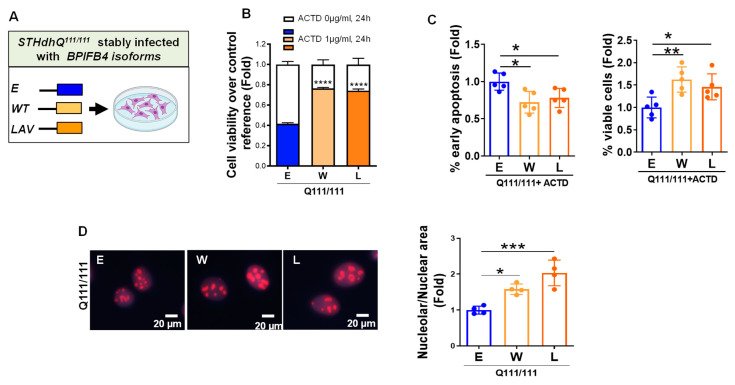
Nucleolar stress in striatal progenitors is mitigated by BPIFB4 isoforms. (**A**–**C**) Effect of nucleolar stress in STHdh^Q111/111^ cells stably infected with BPIFB4 isoforms or empty vector. (**B**) Cytotoxic effects of ACTD (1 μg/mL for 24 h) were analyzed using MTT assay. Graph shows the effects of drug in decreasing viability of STHdh^Q111/111^_E and the mitigation of the cytotoxic effects by BPIFB4 isoforms. N = 3 independent experiments. (**C**) Antiapoptotic effect of BPIFB4 isoforms was analyzed by annexin V-APC/7AAD coupled with flow cytometry. BPIFB4 isoforms reduced the fraction of early-apoptotic cells and increased the rate of cell viability in STHdh^Q111/111^ cells exposed to ACTD (1 µg/mL) for 24 h. N = 5 experiments. (**D**) STHdh^Q111/111^ cells stably infected with BPIFB4 isoforms and empty vector were analyzed for the nucleolar sizes. Nucleoli were stained for NCL (red), and nuclei labelled by Hoechst 33,258 (blue). Graph shows nucleolar shrinkage occurring in STHdh^Q111/111^_E compared to STHdh^Q111/111^ infected with LAV- and a to less extent WT-BPIFB4. N = 4 independent experiments. Data were analyzed using one-way ANOVA (panel **B** and **D**) or unpaired Student’s *t*-test (panel **C**). * *p* < 0.05, ** *p* < 0.01, *** *p* < 0.001, **** *p* < 0.0001.

The immortalized embryonic striatal cell line that carries 111 CAG repeats (STHdh^Q111/Q111^) [25] was stably infected with BPIFB4 isoforms (WT and LAV) and empty vector. No substantial morphological changes have been observed following ectopic BPIFB4 expression. The HD cell model was employed to analyze the nucleolar stress, an emerging element directly involved in HD toxicity [9]. For this purpose, actinomycin D (ACTD), a known transcription inhibitor of RNA polymerase I which interferes with nucleolar homeostasis by promoting a p53-dependent nucleolar stress response, was used [26]. Drug cytotoxicity was assessed by measuring the cell viability through MTT assay. As shown in Figure 1A,B, ACTD significantly (*p* = 0.0001) reduced the viability of empty vector-infected STHdh^Q111/111^ cells (STHdh^Q111/111^_E) by 60%. Next, we challenged the potential protection of BPIFB4 isoforms against toxicity induced by ACTD. Importantly, a modest reduction in the viability, only by 22%, was observed in ACTD-treated and BPIFB4-infected cells. No significant differences in cell viability were observed among untreated cells.

To further validate the antiapoptotic potential of BPIFB4 in ACTD-exposed cells, the reduction of allophycocyanin (APC)-conjugated annexin-v and 7-aminoactinomycin D (7-AAD) double staining, as detected by flow cytometry, was followed as readout. Results indicated a significant (*p* = 0.05) decrease in the distribution of early-apoptotic cells, together with an increase in the fraction of viable cells, in response to ectopic BPIFB4 isoforms (Figure 1C). No effects were observed on the induction of late apoptosis.

Nucleolar morphology is used as a disease biomarker parameter, since the shape, size, and number of the nucleoli may change during diseases and environmental inputs, reflecting alterations in the organelle function [27,28]. Thus, we challenged the putative benefit of BPIFB4 isoforms on the innate toxicity profile of STHdh^Q111/Q111^ cells using as readout the nucleolar morphology analyzed by NCL immunofluorescence staining. As shown in Figure 1D, empty-vector-infected STHdh^Q111/111^ cells displayed smaller nucleoli than those infected with LAV-BPIFB4 and to less extent WT-BPIFB4.

Taken together, these results supported the antiapoptotic effects of BPIFB4 isoforms against nucleolar insults and highlighted a more efficient contribution of LAV-BPIFB4 compared to WT in increasing the nucleolar size, which may translate to improved ribosome biogenesis.

### 2.2. BPIFB4 Isoforms Decrease DNA Damage

**Figure 2:**
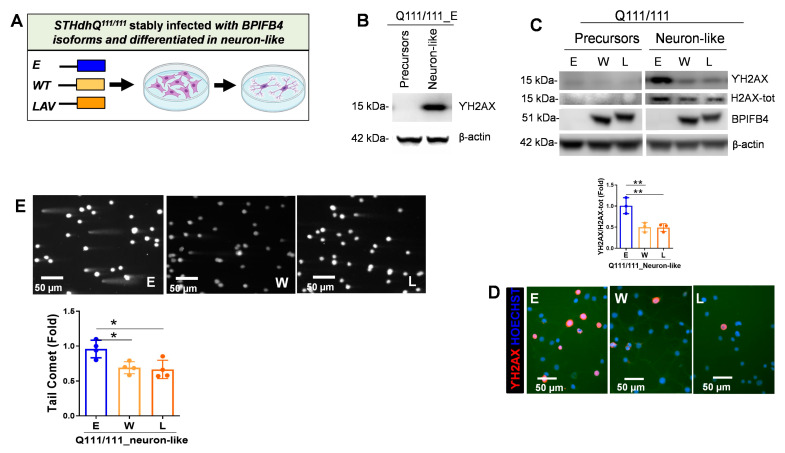
BPIFB4 isoforms decrease the accumulation of DNA damage in neuron-like cells differentiated from striatal precursors. (**A**) Cartoon showing the empty vector- and BPIFB4-infection groups and the differentiative process of striatal precursors into neuron-like cells. (**B**,**C**) Immunoblot detection shows the activation of ΥH2AX in STHdh^Q111/111^ neuron-like cells and its repression in response to the ectopic BPIFB4 isoforms. N = 3 independent experiments. (**D**) Immunofluorescence analysis shows that exogenous BPIFB4 proteins reduced the frequency of ΥH2AX positive cells (ϒH2AX stained in red and nuclei stained in blue). (**E**) Detection of DNA repair efficiency exerted by BPIFB4 isoforms using comet and pulsed-field gel electrophoresis assays. Representative comet images (upper panel), semiquantitative analysis of the results expressed as tail moments (lower panel). N = 4 independent experiments. Data were analyzed using one-way ANOVA. * *p* < 0.05, ** *p* < 0.01.

Several molecular and genetic evidence highlight the interlink between DNA repair dysfunction and HD [13,14,15]. Moreover, DNA double strand breaks (DSBs), characterized by the phosphorylation of H2AX (ΥH2AX), have been reported as an early event in HD primary neurons [15]. In order to make STHdh^Q111/Q111^ cells more similar to HD primary neurons, the precursors were differentiated into neuron-like cells using a well-standardized protocol previously described [25]. As shown in Supplemental Figure S1, compared to precursors, the differentiated cells acquired neuron-like features characterized by elongated branching structures stemming from the cell bodies. Here, we addressed the activation of DNA fragmentation in STHdh^Q111/111^ precursors and in their counterparts induced to differentiate into neuron-like lineage. Immunoblot experiments showed a pronounced activation of ΥH2AX in empty-vector-infected STHdh^Q111/111^ cells following neuronal differentiation (Figure 2A,B), which may be compatible with the susceptibility of mature neurons to DNA damage. Importantly, the observed ΥH2AX induction was drastically blunted in response to BPIFB4 isoform infection (Figure 2C). In accordance, exogenous BPIFB4 proteins led to a drastic decrease in the frequency of the ΥH2AX positive phenotype compared with control (Figure 2D).

To directly evaluate the effect of BPIFB4 isoforms on DNA damage repair efficacy, we then monitored the levels of DNA fragmentation by performing comet and pulsed-field gel electrophoresis assays. A significant (*p* = 0.05) reduction in comet tail length was observed in neuron-like cells overexpressing BPIFB4 isoforms, indicating that these cells were more efficient in resolving DNA damage than empty-vector-infected cells (Figure 2E).

Taken together, the results indicated the protective and reparative effects of BPIFB4 against the DNA damage.

### 2.3. BPIFB4 Isoforms Partially Preserve Cellular Identity

**Figure 3:**
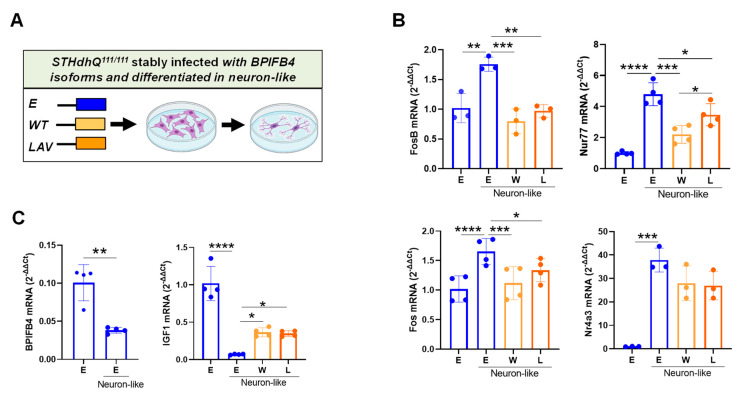
BPIFB4 isoforms partially preserve stemness properties. (**A**) Cartoon showing the empty vector- and BPIFB4-infection groups and the differentiative process of striatal precursors into neuron-like cells. Transcriptional expression of the neuronal activity-regulated genes linked to DNA DSBs (**B**) and stemness genes (**C**) was analyzed by quantitative PCR real-time in neuron-like cells versus progenitors. N = 3 independent experiments. Data were analyzed using one-way ANOVA. * *p* < 0.05, ** *p* < 0.01, *** *p* < 0.001, **** *p* < 0.0001.

Neuronal activity stimulation triggers the formation of DNA DSBs in the promoters of a subset of neuronal early-response genes, which leads to their constitutive expression [29]. Consistently, we observed a significant upmodulation in a group of neuronal activity-regulated genes linked to DNA DSBs (i.e., Fos, FosB, Nur77 and Nr4a3), during the differentiation of empty-vector-infected striatal precursors into neuronal lineage (Figure 3A,B). Interestingly, the observed gene induction was significantly reduced by the ectopic BPIFB4 isoforms (Figure 3B), which may be consistent with their involvement in the partial maintenance of cellular pluripotency. Accordingly, endogenous BPIFB4 and IGF1, a gene playing a crucial role in the long-term proliferation and maintenance of neural stem cells [30], were enriched in the striatal precursors and derepressed in the neuron-like cells (Figure 3C). As consequence, ectopic BPIFB4 isoforms re-augmented the level of IGF1 transcript in neuron-like cells (Figure 3C).

Altogether, the data indicate a role of BPIFB4 in partially counteracting the neuronal differentiation and preserving the cellular identity.

### 2.4. LAV-BPIFB4 Isoform Protects against Apoptosis through the Deregulation of Caspase Cascade

**Figure 4:**
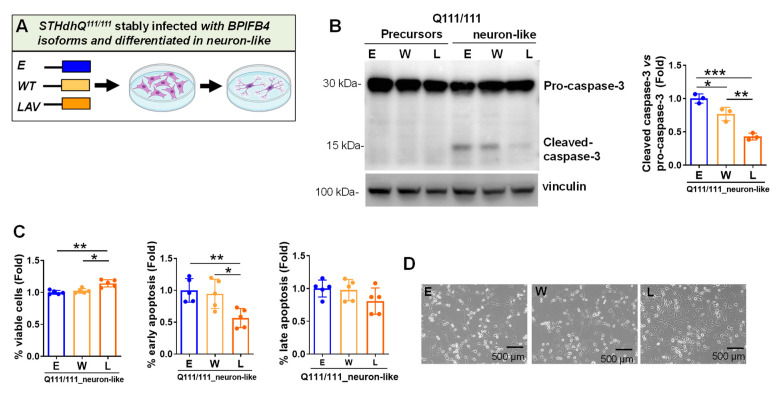
LAV-BPIFB4 isoform counteracts apoptosis via deregulation of caspase 3 cascade. (**A**) Cartoon showing the empty vector control and BPIFB4-infection groups and the differentiative process of striatal precursors into neuron-like cells. (**B**) Immunoblot detection and quantification of cleaved caspase 3 versus pro-caspase 3 show the activation of proapoptotic executor in STHdh^Q111/111^ neuron-like cells and its de-repression in response to the ectopic LAV-BPIFB4 and to lesser extent WT isoform. N = 3 independent experiments. (**C**) Antiapoptotic effect of BPIFB4 isoforms was analyzed by annexin V-APC/7AAD coupled with flow cytometry in STHdh^Q111/111^ neuron-like cells. LAV-BPIFB4 isoform preserved the fraction of viable cells and decreased the percentage of early and late apoptotic cells. WT-BPIFB4 isoform was ineffective. N = 5 experiments. (**D**) Representative images show the morphology of STHdh^Q111/111^ overexpressing BPIFB4 isoforms. Data were analyzed using one-way ANOVA. * *p* < 0.05, ** *p* < 0.01, *** *p* < 0.001.

In HD primary neurons, activation of apoptosis executioner caspase 3 and neuronal death have been reported as a downstream event from the DNA damage [15]. Here, we check if caspase 3 was activated in pathological neuron-like cells and whether this effect can be rescued by BPIFB4 isoforms. As shown in Figure 4A,B, strong activation of caspase 3 was observed during differentiation of empty-vector-infected STHdh^Q111/111^ into neuron-like cells with cleavage inhibition occurring in response to LAV- and, to a lesser extent, WT-BPIFB4 expression (Figure 4B). Next, we challenged the capability of BPIFB4 isoforms to protect against apoptosis, using APC-conjugated annexin-V and 7-AAD double staining coupled with flow cytometry. As shown in Figure 4C, ectopic LAV-BPIFB4 induced a significant (*p* = 0.01) increase in cell viability, accompanied by a global decrease in the rate of apoptosis, which reached statistical significance (*p* = 0.01) for the early stage of apoptosis compared to empty vector and WT-BPIFB4. Consistently, cells overexpressing LAV-BPIFB4 became more closely attached to the substrate than those infected with WT isoform and empty vector (Figure 4D).

Taken together, these results supported the neuro-protective effects of LAV-BPIFB4 against apoptosis through the inactivation of caspase 3 death signaling.

### 2.5. BPIFB4 Isoforms Decrease the Level of Trimethylated Histone 3 on Lysine 9 and Remodel Chromatin

**Figure 5:**
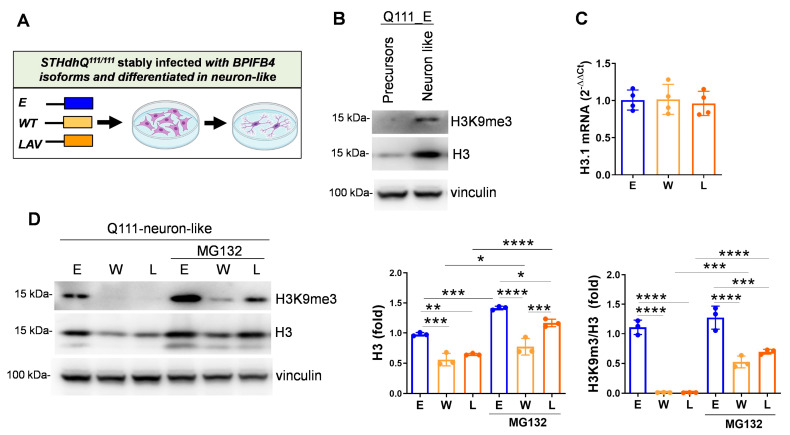
LAV-BPIFB4 balances the aberrant H3K9me3 heterochromatinization via ubiquitin proteasome pathway. (**A**) Cartoon showing the empty vector control and BPIFB4 infection groups and the differentiative process of striatal precursors to neuron-like cells. (**B**) Immunoblot detection of H3K9me3 show that trimethylation occurs in STHdh^Q111/111^ neuron-like cells, N = 3 independent experiments. (**C**) Transcriptional expression of H3.1 was analyzed by quantitative PCR real-time in striatal precursors. BPIFB4 isoforms did not change the histone mRNA levels. (**D**) Immunoblot detection and quantification of H3K9me3 show that trimethylation occurs in STHdh^Q111/111^ neuron-like cells, is rescued by BPIFB4 isoforms and accumulated in response to MG132 treatment. N = 3 independent experiments. Data were analyzed using one-way ANOVA. * *p* < 0.05, ** *p* < 0.01, *** *p* < 0.001, **** *p* < 0.0001.

HD patients and R6/2 transgenic mice are characterized by elevated levels of H3K9me3 that are associated to transcriptional repression and heterochromatinization [11]. Consistently, immunoblot experiments showed the activation of H3K9me3 during the differentiation of empty-vector-infected STHdh^Q111/111^ toward neuron-like lineage, which may reflect the increase of heterochromatinization occurring in mature HD neurons (Figure 5A,B). Of note, a modulation in H3 amount was also observed during neuronal differentiation (Figure 5B). Interestingly, while BPIFB4 isoforms did not impact transcription levels of H3.1 mRNA that codify for H3 (Figure 5C), their overexpression significantly reduced H3 protein levels and to a major extent also methylation of H3K9 (Figure 5D). In order to elucidate whether BPIFB4 targeted H3 and H3K9me3 to degradation through the ubiquitin–proteasomal machinery, cells were treated with the proteasome inhibitor MG132 and analyzed for the accumulation of the substrates by Western blot. As shown in Figure 5D, BPIFB4 isoforms impact on histone availability was partially abolished in response to MG132 treatment.

Taken together, the results indicated that BPIFB4 isoforms target epigenetic histone modification to degrade via the ubiquitin–proteasome system, leading to chromatin remodeling.

HD is characterized by:

1. Nucleolar stress trigged by NCL sequestration in CAG RNA foci
2. DNA damage and DNA repair dysfunction
3. Caspase 3-dependent cell-death pathway
4. H3K9me3-dependent heterochromatin condensation

LAV-BPIFB4:

1. Decreases nucleolar stress
2. Counteracts DNA damage and enhances DNA repair efficiency
3. Inhibits the cleavage of pro-caspase 3, counteracting cell death in favor of cell viability
4. Reduces the level of H3K9me3 through the ubiquitin–proteasome system

## 3. Discussion

The present study provides compelling evidence for a remarkable benefit of LAV, and to lesser extent WT-BPIFB4, in striatal-derived cells expressing human mHTT.

Mechanistic experiments indicate that BPIFB4 protects against nucleolar stress, DNA damage, and heterochromatinization. However, LAV-BPIFB4 exclusively rescued neuron-like cells from apoptosis and was more efficient in preserving the nucleolar size and in targeting the H3 substrates to degrade via ubiquitin–proteosome machinery. These findings complement our previous report regarding the capacity of LAV-BPIFB4 gene therapy to blunt the progression of the disease in a R6/2 HD mouse model, providing new stimulant molecular mechanisms beside the neuroprotective effects exerted by the longevity variant of BPIFB4 [22]. Consistently, our previous work reported a different BPIFB4 isoforms localization. Specifically, LAV-BPIFB4 exhibited a main nuclear distribution while WT-BPIFB4 was more restricted to the perinuclear region [22]. The different observed dynamics between WT- and LAV-BPIFB4 could be account for the more efficacy of LAV-BPIFB4 compared to WT isoform in impacting specific signaling which contributes to ameliorate the HD phenotype.

### 3.1. LAV-BPIFB4 Counteracts the Nucleolar Stress

Our study pinpoints the nucleolus as a subcellular target of the LAV-BPIFB4’s therapeutic action. Notably, mHTT interferes with rDNA transcription and with the integrity of the nucleolus by interacting with NCL, a nucleolar protein that plays multiple roles in rRNA synthesis, ribosome biogenesis and nucleolar structure maintenance [9]. As a consequence, the size, shape and number of nucleoli per cell greatly change, reflecting perturbation in their metabolic functions and activation of the nucleolar stress. This morphological sensitivity to environmental inputs and pathological states makes the nucleolus a potent disease biomarker in several disorders including cardiovascular disease [27,28]. Here, we show that both BPIFB4 isoforms protected striatal precursors expressing mHTT from nucleolar stress through the potentiation of the early antiapoptotic signaling. Nonetheless, LAV-BPIFB4 was more effective than WT-BPIFB4 in preserving the size of the nucleolus.

These functions can be reconducted to the homeostatic properties displayed by LAV-BPIFB4. Data from this study and previous publications indicate that LAV-BPIFB4 restores cell health by activating multiple integrating homeostatic pathways that include the ribosome biogenesis via the increase of small nucleolar RNA (snoRNA), adaptive stress responses through dephosphorylation of eIF2a and proteostasis via the recruitment of eNOS-activating factor HSP90 [17]. The possibility that the LAV-BPIFB4-mediated signaling represents a target for HD treatment was deeper explored in additional dysfunctional mechanisms mediated by nucleolus.

### 3.2. LAV-BPIFB4 Protects HD Neuron-like Cells from DNA Damage and Apoptosis via Inhibition of Caspase Signaling

The nucleolar stress response is further connected to the induction of senescence and DNA damage. Nucleolar proteins represent functional classes involved not only in ribosome biogenesis, but also in DNA-binding and DNA repair. Notably, NCL participated in the DSB-induced DNA damage response, via physical interaction with ΥH2AX and Ku70, a pivotal factor involved in the non-homologous end joining (NHEJ) pathway [31]. Moreover, in NCL-depleted cells, access of repair factors to DSBs is decreased due to the decline in nucleosome disruption mediated by NCL’s histone chaperone activity and its interaction with repair complex [32]. Notably, mHTT activates inappropriate and chronic DNA damage response pathway by interfering with Ku70 and the downstream NHEJ activity [15] that in turn contributes to the HD-related neuronal dysfunctions.

Here, we reported the efficacy of both BPIFB4 isoforms to decrease the protein level of ΥH2AX and the transcripts of a subset of neuronal early-response genes linked to DNA-DSBs that overloaded during the differentiation of striatal precursors into neuron-like phenotype. Likewise, both isoforms augmented the efficiency of DNA repair machinery. The interacting proteins involved in the resolution of DNA repair mediated by BPIFB4 remain largely unknown. NCL and Ku70 may represent potential interactors since both proteins along with BPIFB4 were identified within an interactive multi-protein complex by the immunoprecipitation assay performed by Nuclear Receptor Signaling Atlas consortium (NURSA; http://www.nursa.org accessed on 4 April 2020).

The accumulation of DNA damage is an early event that precedes the apoptotic signal activation in mature neurons expressing mHTT [15]. Consistently, a severe activation of caspase 3 cleavage was observed in the neuron-like cells. In this context, LAV-BPIFB4 displayed the exclusive capacity to rescue neuron-like cells from an early phase of apoptosis via deregulation of caspase 3 signaling and concomitantly improvement of cell viability. The antiapoptotic signaling mediated by LAV-BPIFB4 may represent promising strategies for counteracting the MSN degeneration and loss occurring in HD.

### 3.3. BPIFB4 Isoforms Partially Counteract Neuronal Differentiation

Here we demonstrated that BPIFB4 was upregulated in the embryonic striatal compared to the neuron-like cells and its deregulation in the last might facilitate the differentiative process. In keeping with these findings, BPIFB4 has been found expressed in the hippocampal dentate gyrus, one of the stem-cell-containing niches in the adult mammalian brain [33]. Moreover, we demonstrated that the ectopic expression of BPIFB4 partially counteracted the differentiative process as evinced by the moderate rescue of IGF1 genes and the mild deregulation of neuronal early-response genes. To be noted, changes in the gene expression profile determined by ectopic BPIFB4 represent only a restricted and affected mechanism interfering with the neuronal differentiation which is orchestrated by multiple and complex molecular pathways. Indeed, the ectopic BPIFB4 isoforms do not affected the neuron-like phenotype which is well preserved.

### 3.4. BPIFB4 Reduces the Level of H3K9me3 in HD Neuron-like Cells

Dynamic changes in chromatin structure are a prominent pathological feature of neurodegenerative diseases, progeroid syndromes, cardiac hypertrophy and heart failure [34,35,36,37]. Notably, increased heterochromatin condensation mediated by H3K9 try-methylation correlates with transcriptional dysfunction and neurodegeneration in animal models of HD and human HD patients [10]. Pharmacological treatment with small molecules such as mithramycin, cystamine and nogalamycin reduced the level of H3K9me3 and the associated methyltransferase protein SETDB1, which improved behavior and neuropathological phenotype in HD transgenic mice [10,11,12].

Here, we reported that elevated H3k9me3 heterochromatinization in HD neuron-like cells can be counteracted by BPIFB4 isoforms. Mechanistic investigation indicated that BPIFB4 directed H3K9me3 to degrade through the ubiquitin–proteasome system resulting in the chromatin remodeling. The clearing activity mediated by BPIFB4 may represent a promising approach to target aberrant histone modification and transcription in HD. Moreover, this mechanism may be investigated in other disorders characterized by the accumulation of H3K9me3, including Alzheimer’s disease and heart failure [36,37,38].

Of note, the NURSA consortium identifies in the BPIFB4 multicomplex lysine-specific demethylase 1 (LSD1), which is known to target methylated histone H3K9. Interestingly, recent evidence from studies on a *Drosophila* model of the HD indicates that reduction of Su(var)3-3, the *Drosophila* ortholog of human LSD1, suppressed mHTT induced neurodegeneration [39]. Further investigations may shed light on the potential link between BPIFB4 and LSD1.

## 4. Material and Methods

### 4.1. Cell Maintenance, Treatment, and Differentiation

Immortalized striatal cells (STHdh) derived from a knock-in transgenic mouse containing homozygous Htt loci with a humanized Exon1 with either seven type (STHdh Q^7/7^) or one hundred eleven polyglutamine repeats (STHdh Q^111/111^) were grown at 33 °C in 10% FBS in DMEM (Coriell Institute for Medical Research, Camden, NJ, USA). STHdh Q^111/111^ cells were infected with empty lentiviral vector or particles encoding either WT- or LAV-BPIFB4 as previously described [22].

Cells were treated with ACTD (1 μg/mL for 24 h) (Merk, Lowe, NJ, USA) and MG132 (2.5 μM for 16 h) (Sigma, Kawasaki, Japan) for indicated time.

For differentiation into neuron-like cells, cells were washed once with PBS followed by the addition of the differentiation medium consisting of 10 ng/mL aFGF (Peprotech, Cranbury, NJ, USA), 250 μM IBMX (Sigma), 200 nM PMA (Sigma), 50 μM forskolin (Sigma), and 5 μM dopamine, in serum-free DMEM, as previously described [25]. Cells were incubated in differentiation cocktail for 16 h.

### 4.2. RNA Extraction and Quantitative Real-Time Analysis

RNA was extracted with RNeasy (Qiagen, Germantown, MD, USA), following the protocol provided by the manufacturer. Total RNA concentration and quality were determined using a Nanodrop spectrophotometer (Nanodrop 1000, ThermoFisher, Waltham, MA, USA). Before retro-transcription, DNAse I (ThermoFisher) was employed to remove genomic DNA contamination. Subsequently, Superscript III, Oligo(dT)12-18, dNTPs mix, and RNaseOUT (ThermoFisher) were used to synthesize cDNA, following the manufacturer’s protocol. QuantStudio^TM^ 6 Flex Real-Time PCR System (Applied Biosystems, Waltham, MA, USA) and SYBR Green PCR Master mix (Applied Biosystems, Life Technology) were employed to conduct Real-Time-qPCR analyses, on triplicate samples of retrotranscribed cDNA. Expression levels were normalized to GAPDH. Primer sequences are listed in Supplementary Table S1. Data were expressed as 2^−(ΔΔCt)^.

### 4.3. Western Blotting

Cells were lysed in RIPA buffer containing protease and phosphatase inhibitor cocktails (Sigma-Aldrich, St. Louis, CA, USA). Protein concentration was determined using the Bradford assay (Sigma-Aldrich). Total proteins were separated by electrophoresis using 4–12% NuPAGE Bis-Tris protein gels (Thermo Fisher Scientific, Carlsbad, CA, USA), transferred onto a polyvinylidene difluoride (PVDF) membrane (GE Healthcare, Buckinghamshire, UK), and probed using the conditions indicated in Supplementary Table S2. Blots were revealed by Western Sure Premium Chemiluminescent Substrate LI-COR and by a C-DiGit Blot scanner (LI-COR Biosciences, Lincoln, NE, USA). Densitometric quantifications were normalized relative to beta-Actin signal using Image Studio software (Image Studio Digits Version 4.0.21) (http://www.licor.com, accessed on 2 April 2019).

### 4.4. Immunofluorescence

Cells were seeded onto sterile glass coverslips and fixed with 4% buffered paraformaldehyde (PFA) at room temperature. After blocking, samples were incubated with primary NCL antibody at the dilution 1:1000 (Abcam, Cambrige, UK) and H2AX antibody at the dilution 1:500 (Cell Signaling, Danvers, MA, USA), and revealed with secondary antibodies (Rhodamine-Red anti-rabbit IgG, Jackson Immuno Research, West Grove, PA, USA). Nuclei were counterstained with Hoechst 33,258 and samples were mounted with GelMount aqueous mounting medium (SIGMA). Images were acquired employing an epifluorescence (Leica DMI 6000B, Leica, Wetzlar, Germany). The nucleolar area was measured with ImageJ (NIH) software and calculated for 100 nuclei per condition.

### 4.5. Cell Viability

Cells were plated onto 96-well plates at a density of 2000 cells/well and exposed to ACTD for 24 h. After this time, cells were treated with 3-(4,5-dimethylthiazol-2-yl)-2,5-diphenyltetrazolium bromide MTT (1 mg/mL) (Invitrogen) for 4 h, at 37 °C. Formazan release was quantified at 560 nm, using a Microplate Reader (Biotek, Winooski, VT, USA).

### 4.6. Comet Assay

Cell suspension (10^5^ cells mL^−1^) was then combined with pre-warmed low-melting agarose at a ratio of 1:10 (*v*/*v*) and poured onto the slides. Lysis was performed overnight at 4 °C in an alkaline buffer (2.5 M NaCl, 0.1 M EDTA, 0.01 M Tris, 1% Triton X100, pH 10). Electrophoresis was carried out in 1X Electrophoresis Buffer (0.3 M NaOH, 1 mM EDTA, pH 13) for 45 min at 21 V. After DNA precipitation and wash in 70% ethanol, slides were dried up and DNA stained with SYBR Gold (Thermo-Fisher) before epifluorescence microscopy analysis (Leica DMI 6000B, Leica, Wetzlar, Germany).

Images were analyzed with the OpenComet plugin for ImageJ and tail moment (tail % DNA × tail length) was calculated for 50 cells per condition.

### 4.7. Detection of Apoptosis by Flow-Cytometry

The induction of apoptosis was detected by flow cytometry. Then, 5 × 10^5^ cells were transferred into FACS tube (BD Biosciences, Franklin Lakes, NJ, USA), resuspended in 100 μL 1× annexin-V Buffer (0.1 M Hepes at pH 7.4, 1.4 M NaCl, 25 mM CaCl_2_, BD Biosciences) and stained, for 30 min, at room temperature, with APC-conjugated annexin-V (Immunotools, Friesoythe, Germany) and 7-AAD (BD Biosciences). Unstained samples were used as fluorescence setting controls. Samples were acquired using a BD FACS Fortessa ×20 cell analyzer (BD Biosciences), equipped with 5 lasers. Briefly, following FSC-A/SSC-A morphological setting, based on annexin-V and 7-AAD signal intensity, cells were gated as annexin V^−^/7-AAD^−^ cells (viable), annexin-V^+^/7-AAD^−^ cells (early apoptotic), annexin-V^+^/7-AAD^+^ cells (late apoptotic) and annexin-V^−^/7-AAD^+^ cells (dead). Flow data were analyzed using the BD FACS Diva and the FlowJo v10 software (Tree Star).

## 5. Conclusions

**Figure 6:**
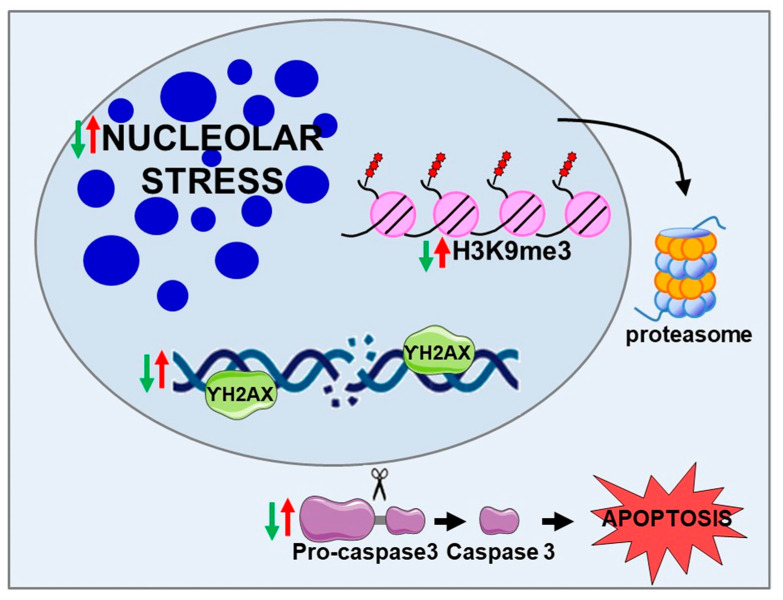
Schematic summarizes altered key mechanisms in HD (red arrows) that are reverted by LAV-BPIFB4 (green arrows).

The nucleolus is a multifunctional organelle with multidimensional roles actively involved in ribosome biogenesis, aging, cell cycle regulation, genome stability, and repair. Proper dynamic control of nucleolar activity is crucial for maintaining tissue homeostasis and health. Alterations in its functions have been associated with cardiovascular and neurodegenerative diseases including HD. In this study, we demonstrated the capacity of longevity variant to correct several dysfunctional mechanisms mainly correlated to nucleolar activity in HD neuron-like cells (Figure 6). In conclusion, LAV-BPIFB4 may represent a promising therapeutic nucleolar modifier of the striking neurodamage occurring in HD.

## Data Availability

The data presented in this study are available in the article.

## Supplementary Materials

The following supporting information can be downloaded at https://www.mdpi.com/article/10.3390/ijms232315313/s1.
