# Supplementary material for: Longevity-Associated Variant of BPIFB4 Confers Neuroprotection in the STHdh Cell Model of Huntington Disease
Source: Int J Mol Sci. 2022 Dec 5;23(23):15313. doi: 10.3390/ijms232315313 (PMC9737551; doi:10.3390/ijms232315313)
Supplement: Supplementary file 1 [file ijms-23-15313-s001.zip › ijms-2082400-supplementary.pdf]

International Journal of *Molecular Sciences*

MDPI

Article

Longevity-Associated Variant of BPIFB4 Confers Neuroprotection in the STHdh Cell Model of Huntington Disease

Monica Cattaneo [1], Anna Maciag [1], Maria Serena Milella [1], Elena Ciaglia [2], Antonino Bruno [3,4] and Annibale Alessandro Puca [1,2,*]

1 Cardiovascular Department, IRCCS MultiMedica, 20138 Milan, Italy
2 Department of Medicine, Surgery and Dentistry "Scuola Medica Salernitana," University of Salerno, 84081 Salerno, Italy
3 Laboratory of Innate Immunity, Unit of Molecular Pathology, Biochemistry and Immunology, IRCCS MultiMedica, 20138 Milan, Italy
4 Laboratory of Immunology and General Pathology, Department of Biotechnologies and Life Sciences (DBSV), University of Insubria, 20138 Varese, Italy
* Correspondence: apuca@unisa.it

Abstract: Huntington's disease

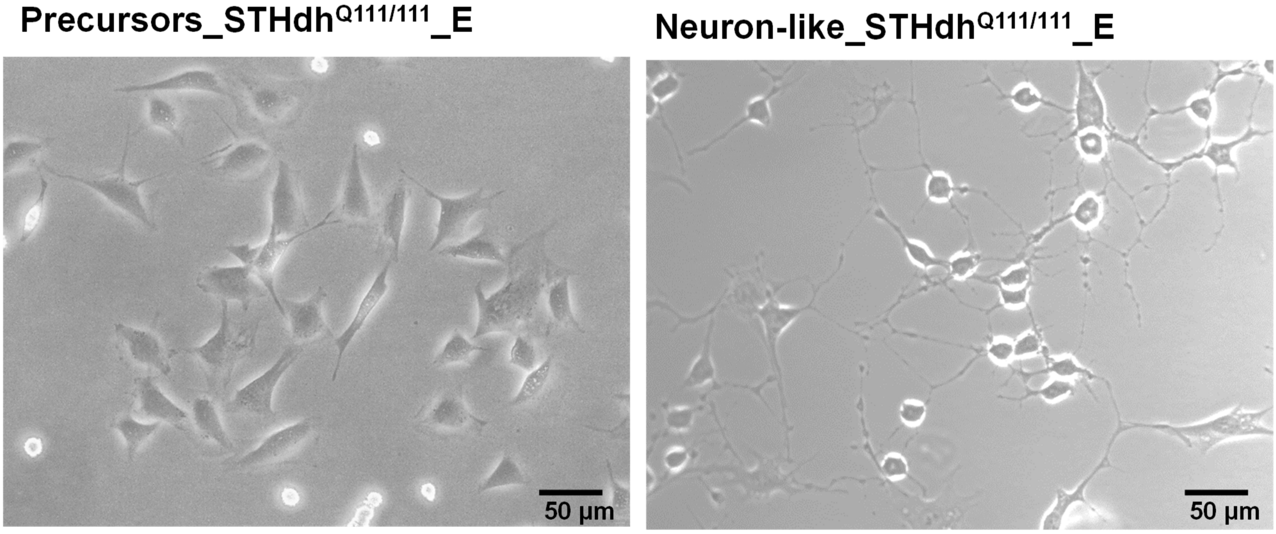

Figure S1. Morphological changes acquired by the STHdhQ111/111_E cells after the differentiation into neuron-like cells. Compared to precursors, the differentiated cells acquired a neuron-like features characterized by elongated branching structures stemming from the cell bodies**.**

Table S1. Primers employed in Realtime RT-PCR experiments.

Gene	Forward	Reverse
**mouse FosB**	ACCTGTCTTCGGTGGACTCCTT	TGGCTGGTTGTGATTGCGGTGA
**mouse Nur77**	GTGCAGTCTGTGGTGACAATGC	CAGGCAGATGTACTTGGCGCTT
**mouse Fos**	GGGAATGGTGAAGACCGTGTCA	GCAGCCATCTTATTCCGTTCCC
**mouse Npas4**	TAGCCCTACTGGACGTTTC	CGGTAGTGTTGAGAAGAAGCTT
**mouse BPIFB4**	CAATTCAGAAGAGTGACGCCT	CGAGGATGAGTCTGCGGCGGAT
**mouse H3.1**	CTGATCCGCAAGCTGCCGTTC	GTTGGTGTCCTCAAACAGACCC

Table S2. Antibodies and experimental conditions employed for Western blot.

ANTIGEN	COMPANY (CATALOG N°)	DILUTION	HOST AND CLONALITY	INCUBATION TIME AND TEMPERATURE	SECONDARY ANTIBODY	INCUBATION TIME
**BPIFB4**	Clinsciences (Custom Made)	1:1000	Rabbit Polyclonal	16 hr, +4°C	ECL Rabbit Igg HPR linked 1:3000	1 hr, TM
**YH2AX**	Biolegend; 613402	1:1000	Mouse Monoclonal	16 hr, +4°C	ECL Mouse Igg HPR linked 1:3000	1 hr, TM
**H2AX**	Cell Signaling; 7631	1:1000	Mouse Monoclonal	16 hr, +4°C	ECL Mouse Igg HPR linked 1:3000	1 hr, TM
**Caspase-3**	Cell Signaling; 14220	1:1000	Rabbit Polyclonal	16 hr, +4°C	ECL Rabbit Igg HPR linked 1:3000	1 hr, TM
**H3K9me3**	Abcam; ab8898	1:500	Rabbit Polyclonal	16 hr, +4°C	ECL Rabbit Igg HPR linked 1:3000	1 hr, TM
**H3**	Abcam; ab1791	1:1000	Rabbit Polyclonal	16 hr, +4°C	ECL Rabbit Igg HPR linked 1:3000	1 hr, TM
**β-actin**	Abcam; ab6276	1:3000	Mouse Monoclonal	16 hr, +4°C	ECL Mouse Igg HPR linked 1:3000	1 hr, TM
**Vinculin**	Sigma; V9131	1:1000	Mouse Monoclonal	16 hr, +4°C	ECL Mouse Igg HPR linked 1:3000	1 hr, TM
